# The presence of overlapping quality of life symptoms in primary antibody deficiency (PAD) and chronic fatigue syndrome (CFS)

**DOI:** 10.1186/s13223-020-0417-3

**Published:** 2020-03-30

**Authors:** Rhea A. Bansal, Susan Tadros, Amolak S. Bansal

**Affiliations:** 1 Frimley Health NHS Trust, Portsmouth Road, Camberley, Surrey GU16 7UJ UK; 2 The Royal Free Hospital, Pond Street, Hampstead, London, NW3 2QG UK; 3 St Anthony’s Hospital, 801 London Road, Cheam, SM3 9DW UK

**Keywords:** Primary antibody deficiency (PAD), Chronic fatigue syndrome (CFS), Quality of life (QoL)

## Abstract

**Background:**

Fatigue, sleep disturbance and altered mood are frequently reported in patients with primary antibody deficiency syndrome (PADS) on adequate immunoglobulin replacement therapy. This study aimed to determine the frequency of symptoms compatible with chronic fatigue syndrome (CFS) in patients with PADS.

**Methods:**

The study involved the distribution of 682 self-completed postal questionnaires to ascertain the presence and frequency of symptoms compatible with CFS in patients with PADS. The reporting of symptoms for each patient were scored against the CFS diagnostic criteria used within our own South London Chronic Fatigue service.

**Results:**

The frequency of symptoms compatible with CFS were evident in 26 of the 188 patients (16.25%) returning adequately completed questionnaires. We considered a bias in the return of questionnaires amongst PADS patients with fatigue to be likely. As such we estimated the minimum frequency of CFS in patients with PADS to be 4% based on the 682 PAD patients to whom the questionnaire was distributed. This was significantly higher than the 0.5% estimate of the prevalence of CFS in the community in western populations. While the presence of significant fatigue correlated with the presence of anxiety and depression, there was no association with self-reported lung damage. Sleep disturbance affected 60% of the PAD patients returning satisfactory questionnaires and as expected the CFS score was higher in those with greater physical limitation.

**Conclusions:**

We conclude that patients with PADS have a high frequency of fatigue, low mood and anxiety. We suggest routine questioning for the symptoms of fatigue, disturbed sleep and altered mood in patients with PADS. The use of several treatment strategies in CFS may prove beneficial in improving the quality of life of patients with PAD.

## Background

Chronic fatigue syndrome (CFS) is a clinical diagnosis based on over 6 months of new and significant fatigue that is not relieved by adequate rest and sleep and which is exacerbated in a delayed fashion by any physical, mental and emotional over-activity. It is frequently accompanied by non-restorative sleep, arthralgia, myalgia, autonomic dysfunction, headaches, hypersensitivity to lights and sounds and impaired concentration and short term memory. Many patients date the onset of their symptoms after a viral or other infection and often during a period of increased psychosocial stress. Routine tests for haematological and biochemical dysfunction, endocrinopathy, inflammation, autoimmunity and immune deficiency are normal and there are presently no diagnostic tests. Indeed, diagnosis is frequently based on patients fulfilling the Canadian [1], international [2] or institute of medicine [3] CFS/ME criteria, with normal blood tests and in the absence of a medical or psychological cause of the fatigue. Interestingly, patients with CFS frequently report recurrent infections and especially upper respiratory tract symptoms such as sore throat with associated cervical lymphadenopathy as well as recurrent ‘colds’ or viral-type symptoms. Importantly, the presence of these symptoms forms part of the diagnostic criteria for CFS [4, 5]. We have previously found significant abnormalities of B cells [6] and T cells [7] in patients with CFS who otherwise had normal serum immunoglobulins. This included increased numbers of naïve B cells and transitional B cells but reduced numbers of plasmablasts [6]. There is also a significant literature suggesting a subtle impairment of cellular immune function in patients with CFS [8, 9].

Primary antibody deficiency (PAD) is diagnosed when symptomatic recurrent infections affecting especially the upper and lower respiratory tract and needing frequent antibiotics are combined with reduced immunoglobulins or IgG subclasses and in the presence of impaired specific IgG responses to common vaccines/antigens. There are several reports on specific criteria for diagnosing PAD but all have the previous broad definition in mind [10, 11]. Many patients with PAD have considerable issues with their physical health which, together with unemployment, have been shown to contribute significantly to a reduced quality of life (QoL). In addition, patients with PAD also experience significant psychosocial and economic distress [12]. It has been widely documented that patients with PAD have reduced perceived health and poorer QoL than healthy matched controls [13]. This was evident in both adults [14, 15] and in children [16–18]. In the case of children with primary immune deficiency, their QoL was considered comparable to that of patients with cancer [17]. Importantly, the reduced QoL in PAD patients is likely due to both physical and psychosocial factors [19].

While earlier diagnosis and commencement of appropriate immunoglobulin replacement therapy has done much to improve physical health and reduce the frequency of infections [20], there remains a significant reduction in QoL that is related to the number of illness-related health conditions experienced by the patient [14, 16, 17]. In addition, the ‘life quality index’ was better in some patient populations on home therapy as opposed to hospital based immunoglobulin replacement therapy [21]. Interestingly, one of the main predictors of QoL in virtually all chronic conditions is fatigue. This was reported to affect 25.9% of PAD patients compared to 6.4% of those with primary immunodeficiency not principally affecting or confined to the antibody system e.g. di George’s syndrome, chronic granulomatous disease, severe combined immunodeficiency. Indeed, the frequency of fatigue in this latter group was similar to the US general population [22]. Interestingly, fatigue in the PAD patients was more frequent in women, those with depression, bronchiectasis, higher body mass index, autoimmunity and those with Common Variable Immune Deficiency (CVID) as opposed to XLA and miscellaneous antibody deficiency [22].

We have noted that many of our patients with PAD have significant chronic fatigue which adversely impacts their mental and physical health, as well as their daily living. The fatigue has similarities to that seen in our patients with CFS-particularly the delayed post-exertional malaise that characterises CFS [23]. Our earlier unpublished work between 2002 and 2006 had shown fatigue and mood disturbance to be frequent in those with PAD and unrelated to frequent infections or lung damage. We therefore hypothesised that patients with symptoms compatible with a diagnosis of CFS/ME are increased in PADS and without any association with lung damage. We sought to determine this using a self-completed questionnaire distributed to PADS patients known to the UK PID registry. Our results suggest that there is indeed an increased frequency of CFS/ME symptoms in those with PADS which may impact significantly in these patients and which may benefit from the treatment strategies used in those with CFS/ME.

## Methods

Modified CFS questionnaires were sent by the ‘Primary Immunodeficiency Association’ to patients with PAD on the PIA register and initial details on the study were provided via their newsletter. Patients were recruited from the 47 Immunology centres across the UK between 2008 and 2009. All patients had been on treatment for longer than 6 months. The questionnaire consisted of 27 questions relating to aspects of mood, sleep, autonomic neural hypersensitivity (intolerance of light/sound, sensation of dizziness on standing, headaches), all of which required binary yes/no responses. Patients were asked to consider their health and symptoms over the entire infusion cycle and not just in relationship to the few days before and after their infusion. Many of these questions were related to the core symptoms recognised in CFS and based on the questionnaire sent to patients with suspected CFS seen in the Sutton CFS service. This utilises a scoring system for confirming the diagnosis of CFS in patients presenting with chronic fatigue without an underlying physical and mental cause (Table 1). A score of 8 or more out of 13 for the core symptoms of CFS in the presence of normal blood tests for haematological, endocrine, renal, hepatic and immune abnormalities and with negative tests for inflammation, gluten sensitivity and autoimmunity was highly suggestive/diagnostic of CFS [23]. This scoring system was applied to the fatigue questionnaire used in the present survey to see how many patients within our cohort fulfilled our diagnostic criteria for CFS.

**Table 1:** CFS diagnostic questionnaire. Source: [23]

Factor	Score
Delayed prolonged post-exertion malaise after increases in physical, mental and emotional activity	3
Non-restorative sleep with frequent difficulty initiating and/or maintaining sleep	2
Impaired concentration that is reduced further by external stimuli	1
Reduced short term memory with word finding difficulty	1
New onset headaches (> 2/month and different in character from previous headaches)	1
Sore throat with cervical tenderness/recurrent flu-like episodes	1
Arthralgia affecting several joints with stiffness > 1 h but no swelling	1
Myalgia affecting multiple groups and exacerbated by mild exertion	1
Postural instability feeling unstable on standing, prolonged standing or sitting	1
Hypersensitivity to sounds and lights (smells and to a lesser degree taste also)	1

Maximum score 13. A score of 8 or more is required for a diagnosis of CFS to be made

Patients were also asked if they had lung damage confirmed on x-ray or CT imaging. These details were confirmed by contact with the respective immunology department involved in their care. Finally, we asked them to quantify their exercise tolerance. This was assessed by the following question: “How far can you comfortably walk in 20 min?”. Responses were graded as ‘severe’, ‘moderate’, ‘mild’ or ‘normal’. Definitions were established based on the average walking speed of 5 km/h and were as follows:

- Severe: wheelchair user/disabled/able to walk up to 500 m in 20 min.
- Moderate: able to walk between 500 and 800 m in 20 min.
- Mild: able to walk 800 m–1.5 km in 20 min.
- Normal: no limitation, able to walk for more than 1.5 km in 20 min.

Finally, the study also aimed to determine the frequency of symptoms of anxiety, depression and sleep disturbance in PAD and to see if there was a correlation between these variables and the CFS score.

## Results

A total of 680 questionnaires were issued of which 211 were returned including 23 which had incomplete information. This left 188 satisfactorily completed questionnaires which form the basis of our report (Table 2). The majority of patients that completed the questionnaire had CVID (162 patients, 86%) with the remainder having X-linked agammaglobulinaemia (12 patients—6%) and defects of specific antibody production (4 patients, 2%). Over a third of patients had radiographic evidence of lung damage (Table 2).  All of the 188 patients were on immunoglobulin replacement therapy which in the UK is only offered on the NHS to patients who fulfil the criteria for PAD based on extensive testing [10, 11]. Sixty percent of the 188 patients were on SCIG and the remaining 40% were on IVIg. At the time of this survey, the number of patients on home immunoglobulin therapy was similar to those receiving their treatment in hospital.

**Table 2:** Patient demographics

Number of patients	188
Mean age (interquartile range)	52 (4–89)
Females (%)	116 (61.7)
Evidence of lung damage on XR/CT (%)	69 (37.3)

**Fig. 1:**
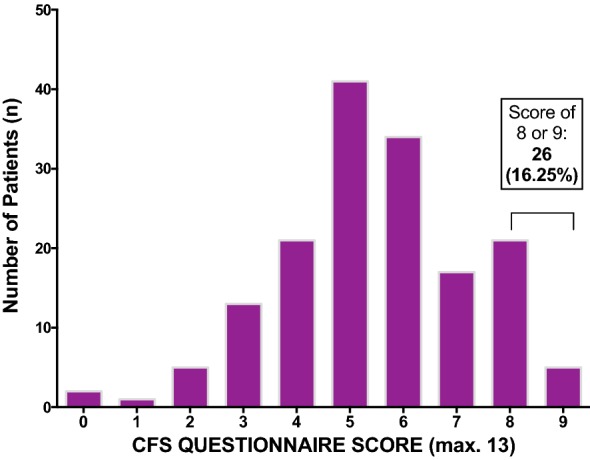
Symptom scoring of PAD patients using the CFS questionnaire

Our results show that a significant proportion of patients with PAD had symptoms compatible with a diagnosis of CFS (Fig. 1). All the symptoms that feature in the CFS diagnostic scoring system were reported in our patient group with a frequency varying between 40 and 90%. One-sixth of the PAD patients (16.3%) scored 8 or more on the CFS questionnaire (the diagnostic threshold for CFS), with a further 31.9% of patients having borderline scores (6–7/13). There was no clear difference in the frequency of CFS symptoms in those on SCIG versus those on IVIg and those on home therapy versus those receiving their treatment in hospital.

**Fig. 2:**
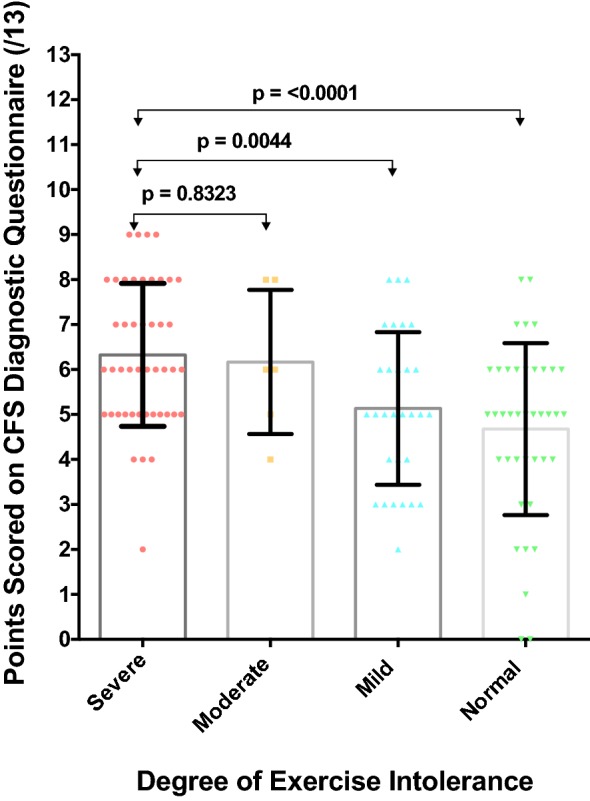
Association between exercise tolerance and points attained on the CFS questionnaire

**Fig. 3:**
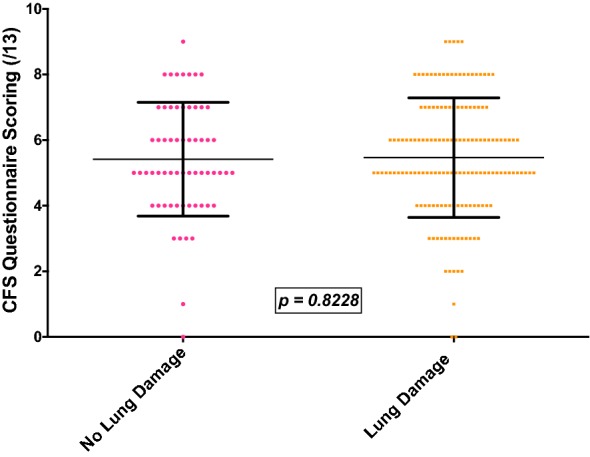
Association between the presence of radiologically-confirmed lung damage and CFS questionnaire scores

As would be expected, the mean CFS score of patients with severe vs. normal exercise intolerance was significantly higher at 6.3 vs. 4.7 (p < 0.0001) (Fig. 2). Thus, maintaining some degree of fitness appears to be associated with a reduced frequency of symptoms associated with CFS. Surprisingly, there was no difference with the mean CFS questionnaire score in patients with reported lung damage vs. no lung damage (5.5 vs. 5.4, p = 0.802) (Fig. 3). Thus, lung damage with underlying infection and inflammation appears not to be linked to fatigue and the mechanism for the latter is more complex and likely multifactorial.

**Fig. 4:**
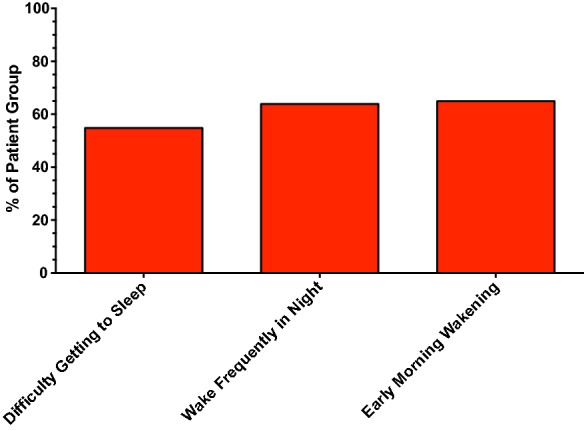
Presence of symptoms related to sleep disturbance

**Fig. 5:**
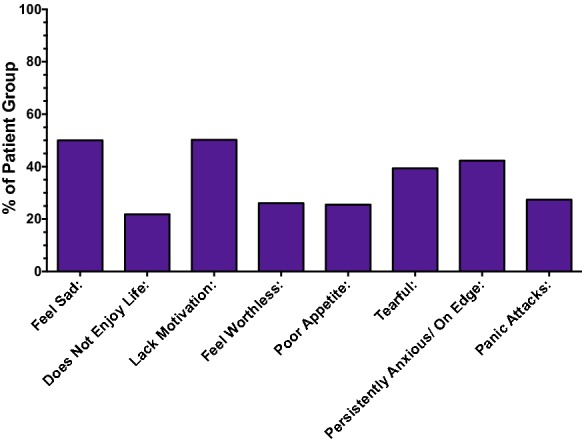
Presence of symptoms related to anxiety and depression

With regards to the main factors contributing to fatigue, 61.2% of patients reported at least one symptom related to sleep disturbance. 35.3% of patients reported at least one symptom related to depression and anxiety (Figs. 4 and 5, respectively). Interestingly, having lung damage seemed to be associated with a fewer number of reported symptoms related to depression (p = 0.034), although the reason for this is unclear.

**Fig. 6:**
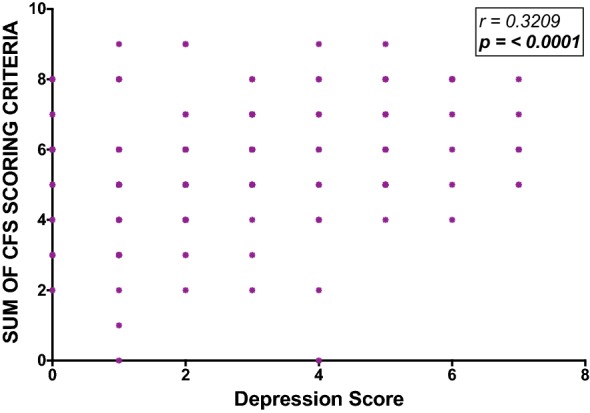
Correlation between overall CFS questionnaire scores and depression symptom scoring

**Fig. 7:**
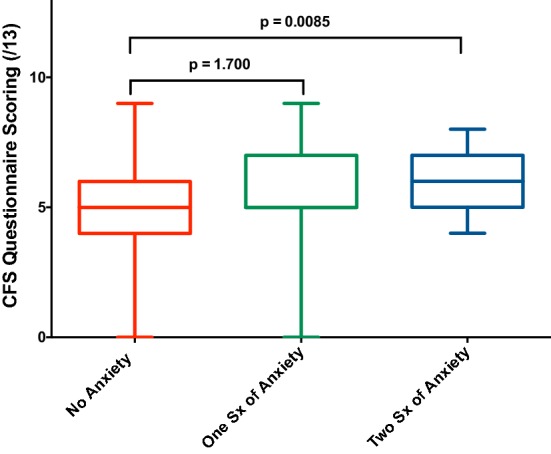
Anxiety symptom scoring and overall CFS questionnaire scores

Having an increasing number of symptoms associated with depression was positively correlated with the CFS score; depression r = 0.32, p < 0.0001 (Fig. 6). There was also a significant increase in the CFS questionnaire score with a coexistent increased severity of anxiety (Fig. 7). As such the mean CFS score with no symptoms of anxiety present vs. 2 symptoms present was significantly different (5.1 vs. 6.1, p = 0.0085).

## Discussion

Although fatigue can affect a quarter of patients with PAD and is particularly prevalent in patients with CVID [22], this is the first study investigating the frequency of symptoms compatible with CFS in patients with PAD. There were circa 2300 patients on the UK primary immune deficiency (UKPIN) database in 2012 [24]. Of these patients 1364 had a diagnosis of PAD and just under half of these (i.e. 680) were on the UK PIN register at the time of this study and were therefore contacted. However, the 188 satisfactorily completed questionnaires represents only a small proportion of all patients with PAD who could have participated in this study. The factors that may have encouraged some patients, and not others, to participate in this type of self-completed questionnaire study is unclear but a selection bias is likely and it is highly probable that those returning their questionnaires were those experiencing more significant fatigue.

Nearly half of all the patients had significant fatigue with CFS scores of over 6. However, 26 out of the 188 PAD patients had a score of 8 or more and therefore met the diagnostic threshold for a diagnosis of CFS. This would suggest a minimum prevalence of CFS in the PAD patients of about 4%; 26 from a potential 680 contactable patients with PAD. This is certainly higher than the 0.5% average prevalence of CFS in the general population in the USA [25]. Thus, having a PAD significantly increases the chances of suffering CFS associated symptoms. However, it is unclear whether this would be related to the underlying immune deficiency or the psychosocial burden of a chronic illness with an increased susceptibility to recurrent infections. Our finding hints indirect and very tentative support for the notion that patients with CFS, who have reduced NK cell numbers and altered B and T cell maturation [6, 7], may have some subtle immune dysfunction contributing or caused by their illness. However, this would be of insufficient severity to produce recurrent pneumonia, sinusitis, meningitis etc. and routine tests of immune function are almost invariably normal [23].

The absence of a significant difference in the frequency of fatigue in those with and without self-reported lung damage appears to suggest that infection and impaired lung function per se may not be an important contributing factor for the development of fatigue. The higher CFS score in those with progressively greater exercise intolerance is likely to be more complex than a simple direct correlation between fatigue and reduced exercise tolerance. The questionnaire unfortunately did not determine the cause of the reduced exercise tolerance. Factors such as shortness of breath, muscle weakness or the post-exertional malaise may be involved. Post-exertional malaise is a characteristic feature of CFS that often results in reduced physical activity. Exploration of the factors involved in reduced exercise tolerance would be an interesting area to investigate further in future studies.

The main aim of this study was to investigate the frequency of CFS compatible symptoms in those with PAD. As such we did not focus on the type of antibody replacement therapy received. Nevertheless, there was no significant difference in the CFS score in those on home versus hospital-based therapy and also those receiving SCIG versus those on IVIg. This would appear to suggest that the PAD itself is contributing directly to the chronic fatigue and is not especially modified by the route or location of replacement therapy.

Symptoms characteristic of depression were frequent in the PAD patients. This contrasts with the report by Heath et al. [26], who reported a similar prevalence of depression within their cohort to the general US population. Depression is well known to be associated with chronic fatigue and is seen not infrequently in patients with CFS [27–29]. Interestingly, many patients with CFS say that their low mood and depression are the result of their chronic unrelenting and unpredictable fatigue and not caused by it. Regardless, actively seeking and treating depression in patients with PAD is suggested as this may improve overall QoL and perhaps chronic fatigue. Regardless, measures to increase physical fitness using a flexible and gradually introduced muscle strengthening regime may also be helpful. In patients with CFS, graded exercise therapy has been shown to be helpful [30] and without major risk of disease relapse. However, the correct starting point of the therapy, along with careful monitoring of the rate of increase of the exercise regime is, in our opinion, critical to prevent relapse and worsening fatigue. Improperly used GET may cause harm and clinicians should be alert to this possibility if highly fatigued patients with PAD are commenced on inadequately monitored exercise therapy.

Many of the patients who participated in this study reported anxiety. The questionnaire did not explore the reasons for this but previous work suggests the importance of frequency and unpredictability of infections as prime factors. Efforts to reduce anxiety may help to reduce overall fatigue and improve QoL. Indeed, the use of cognitive behavioural therapy was previously found to be helpful in 44 PAD patients who noted improvements levels of fatigue as well as anxiety, depression and insomnia [31]. The authors noted a high level of acceptability of the service and suggested “the potential for long-term cost saving to the NHS”.

Over half of the PAD patients that participated reported some aspect of sleep disturbance; particularly unrefreshing or non-restorative sleep. The latter is frequent in those with CFS [32, 33] and while the precise cause of the sleep disturbance in CFS is unclear, it is not thought to be particularly correlated with previous levels of activity and exertion [34]. Nonetheless, treating insomnia and sleep disturbance has been shown to be beneficial for the fatigue in CFS [35]. As such attention to uncovering and treating sleep disturbance is recommended in patients with PAD with a view to improving their fatigue and overall QoL.

## Conclusion

Overall our work has demonstrated that there is a high frequency of several symptoms in patients with PAD which collectively are seen in patients diagnosed with CFS and which may contribute to a lower QoL. It is important to routinely enquire about fatigue, sleep disturbance and symptoms of depression and anxiety when reviewing patients with PAD. Early identification and treatment of these features, together with consideration of utilising strategies currently used in the management of CFS may help to improve the QoL of this group of patients. The mechanism of the fatigue in PAD is likely multifactorial and in part related to low mood, anxiety, sleep disturbance and limitation of physical activity [36] imposed by the underlying immune defect and the subsequent infections. Recurrent infection and the unpredictability and vulnerability to infection in patients with PAD is likely to produce chronic emotional, inflammatory, nitrosative and oxidative stress which is well known to be associated with chronic fatigue. Here there is similarity to the situation in CFS [37]. We speculate whether the collective stress experience in patients with PAD and related to all the above factors may also aggravate any tendency to impaired cellular immunity. The latter may already be compromised in a subset of patients with PAD and especially those with CVID. Regardless, cellular immunity normally prevents the reactivation of the common herpes type viruses such as EBV, CMV and HHV6 that are known to be associated with fatigue and several of the other symptoms seen in CFS [38]. Heightened viral reactivation may therefore contribute to the fatigue seen in patients with PAD which was previously found to be greater in CVID compared to XLA [22]. Research correlating tests of cellular immunity with herpes viral copy numbers and fatigue is suggested to investigate this possibility further.

## Data Availability

All authors agree for the manuscript to be made freely available. The datasets generated and/or analysed during the current study are not publicly available but are available from the corresponding author on reasonable request.

## References

[1] Carruthers BM (2007). Definitions and aetiology of myalgic encephalomyelitis: how the Canadian consensus clinical definition of myalgic encephalomyelitis works. J Clin Pathol.

[2] Carruthers BM, van de Sande MI, De Meirleir KL (2011). Myalgic encephalomyelitis: international consensus criteria. J Intern Med.

[3] Committee on the Diagnostic Criteria for Myalgic Encephalomyelitis/Chronic Fatigue Syndrome; Board on the Health of Select Populations; Institute of Medicine. Washington (DC): Beyond Myalgic Encephalomyelitis/Chronic Fatigue Syndrome: Redefining an Illness. National Academies Press (US); 2015. The National Academies Collection: Reports funded by National Institutes of Health. 25695122

[4] Fukuda K, Straus SE, Hickie I, Sharpe MC, Dobbins JG, Komaroff A (1994). The chronic fatigue syndrome: a comprehensive approach to its definition and study. International Chronic Fatigue Syndrome Study Group. Ann Intern Med.

[5] Carruthers BM, van de Sande MI, De Meirleir KL, Klimas NG, Broderick G, Mitchell T (2011). Myalgic encephalomyelitis: international consensus criteria. J Intern Med.

[6] Bradley AS, Ford B, Bansal AS (2013). Altered functional B cell subset populations in patients with chronic fatigue syndrome compared to healthy controls. Clin Exp Immunol.

[7] Ford B, Bradley AS, Bansal AS (2016). Altered functional T cell subset populations and cytokine profile in patients with chronic fatigue syndrome: a pilot study. J Chronic Dis Manag.

[8] Mensah FKF, Bansal AS, Ford B, Cambridge G (2017). Chronic fatigue syndrome and the immune system: where are we now?. Neurophysiol Clin.

[9] Bansal AS, Bradley AS, Bishop KN, Kiani-Alikhan S, Ford B (2012). Chronic fatigue syndrome, the immune system and viral infection. Brain Behav Immun.

[10] Filion CA, Taylor-Black S, Maglione PJ, Radigan L, Cunningham-Rundles C (2019). Differentiation of common variable immunodeficiency from IgG deficiency. J Allergy Clin Immunol Pract.

[11] Jolles S, Chapel H, Litzman J (2017). When to initiate immunoglobulin replacement therapy (IGRT) in antibody deficiency: a practical approach. Clin Exp Immunol.

[12] Guani-Guerra E, Jimenez-Romero AI, Garcia-Ramirez UN, Velazquez-Avalos JM, Martinez-Guzman E, Sandoval-Ramirez E (2017). Disease burden for patients with primary immunodeficiency diseases identified at reference hospitals in Guanajuato, Mexico. PLoS ONE.

[13] Seeborg FO, Seay R, Boyle M, Boyle J, Scalchunes C, Orange JS (2015). Perceived health in patients with primary immune deficiency. J Clin Immunol.

[14] Barlogis V, Mahlaoui N, Auquier P, Pellier I, Fouyssac F, Vercasson C (2017). Physical health conditions and quality of life in adults with primary immunodeficiency diagnosed during childhood: a French Reference Center for PIDs (CEREDIH) study. J Allergy Clin Immunol.

[15] Rider NL, Kutac C, Hajjar J, Scalchunes C, Seeborg FO, Boyle M (2017). Health-related quality of life in adult patients with common variable immunodeficiency disorders and impact of treatment. J Clin Immunol.

[16] Ataeinia B, Montazeri A, Tavakol M, Azizi G, Kiaee F, Tavakolinia N (2017). Measurement of health-related quality of life in primary antibody-deficient patients. Immunol Invest.

[17] Sultan S, Rondeau E, Levasseur M-C, Dicaire R, Decaluwe H, Haddad E (2017). Quality of life, treatment beliefs, and treatment satisfaction in children treated for primary immunodeficiency with SCIg. J Clin Immunol.

[18] Titman P, Allwood Z, Gilmour C, Malcolmson C, Duran-Persson C, Cale C (2014). Quality of life in children with primary antibody deficiency. J Clin Immunol.

[19] Aghamohammadi A, Montazeri A, Abolhassani H, Saroukhani S, Pourjabbar S, Tavassoli M (2011). Health-related quality of life in primary antibody deficiency. Iran J Allergy Asthma Immunol.

[20] Kearns S, Kristofek L, Bolgar W, Seidu L, Kile S (2017). Clinical profile, dosing, and quality-of-life outcomes in primary immune deficiency patients treated at home with immunoglobulin G: data from the IDEaL patient registry. J Manag Care Spec Pharm.

[21] Bienvenu B, Cozon G, Hoarau C, Pasquet M, Cherin P, Clerson P (2016). Does the route of immunoglobin replacement therapy impact quality of life and satisfaction in patients with primary immunodeficiency? Insights from the French cohort “Visages”. Orphanet J Rare Dis.

[22] Hajjar J, Guffey D, Minard CG, Orange JS (2017). Increased incidence of fatigue in patients with primary immunodeficiency disorders: prevalence and associations within the US immunodeficiency network registry. J Clin Immunol.

[23] Bansal AS (2016). Investigating unexplained fatigue in general practice with a particular focus on CFS/ME. BMC Fam Pract.

[24] Edgar JDM, Buckland M, Guzman D, Conlon NP, Knerr V, Bangs C (2014). The United Kingdom Primary Immune Deficiency (UKPID) Registry: report of the first 4 years’ activity 2008–2012. Clin Exp Immunol.

[25] Centers for Disease Control and Prevention. What is ME/CFS?|Myalgic Encephalomyelitis/Chronic Fatigue Syndrome (ME/CFS)|CDC. 2018. https://www.cdc.gov/me-cfs/about/index.html. Accessed 23 June 2019.

[26] Heath J, Lehman E, Saunders EFH, Craig T (2016). Anxiety and depression in adults with primary immunodeficiency: how much do these patients experience and how much do they attribute to their primary immunodeficiency?. Allergy Asthma Proc.

[27] Krupp LB, Sliwinski M, Masur DM, Friedberg F, Coyle PK (1994). Cognitive functioning and depression in patients with chronic fatigue syndrome and multiple sclerosis. Arch Neurol.

[28] Mariman A, Delesie L, Tobback E, Hanoulle I, Sermijn E, Vermeir P (2013). Undiagnosed and comorbid disorders in patients with presumed chronic fatigue syndrome. J Psychosom Res.

[29] Nater UM, Lin J-MS, Maloney EM, Jones JF, Tian H, Boneva RS (2009). Psychiatric comorbidity in persons with chronic fatigue syndrome identified from the Georgia population. Psychosom Med.

[30] Larun L, Brurberg KG, Odgaard-Jensen J, Price JR (2017). Exercise therapy for chronic fatigue syndrome. Cochrane Database Syst Rev.

[31] Campbell M, Clarke A, Symes A, Workman S, Stauss H, Webster AD (2018). Investigating the effectiveness, acceptability and impact on healthcare usage of providing a cognitive-behavioural based psychological therapy service for patients with primary antibody deficiency. J Clin Immunol.

[32] Russell C, Wearden AJ, Fairclough G, Emsley RA, Kyle SD (2016). Subjective but not actigraphy-defined sleep predicts next-day fatigue in chronic fatigue syndrome: a prospective daily diary study. Sleep.

[33] Josev EK, Jackson ML, Bei B, Trinder J, Harvey A, Clarke C (2017). Sleep quality in adolescents with chronic fatigue syndrome/myalgic encephalomyelitis (CFS/ME). J Clin Sleep Med.

[34] Rahman K, Burton A, Galbraith S, Lloyd A, Vollmer-Conna U (2011). Sleep–wake behavior in chronic fatigue syndrome. Sleep.

[35] Kallestad H, Jacobsen HB, Landro NI, Borchgrevink PC, Stiles TC (2015). The role of insomnia in the treatment of chronic fatigue. J Psychosom Res.

[36] Sowers KL, Litwin BA, Lee ACW, Galantino MLA (2018). Exercise perception and behaviors in individuals living with primary immunodeficiency disease. J Clin Immunol.

[37] Morris G, Maes M, Berk M, Puri BK (2019). Myalgic encephalomyelitis or chronic fatigue syndrome: how could the illness develop?. Metab Brain Dis.

[38] Morris G, Berk M, Walder K, Maes M (2016). The putative role of viruses, bacteria, and chronic fungal biotoxin exposure in the genesis of intractable fatigue accompanied by cognitive and physical disability. Mol Neurobiol.

